# Sulfated vizantin causes detachment of biofilms composed mainly of the genus *Streptococcus* without affecting bacterial growth and viability

**DOI:** 10.1186/s12866-020-02033-w

**Published:** 2020-11-25

**Authors:** Taisuke Hasegawa, Shoji Takenaka, Masataka Oda, Hisanori Domon, Takumi Hiyoshi, Karin Sasagawa, Tatsuya Ohsumi, Naoki Hayashi, Yasuko Okamoto, Hirofumi Yamamoto, Hayato Ohshima, Yutaka Terao, Yuichiro Noiri

**Affiliations:** 1 Division of Cariology, Operative Dentistry and Endodontics, Faculty of Dentistry & Graduate School of Medical and Dental Sciences, Niigata University, 2-5274, Gakkocho-dori, Chuo-ku, Niigata, 951-8514 Japan; 2 Department of Microbiology and Infection Control Science, Kyoto Pharmaceutical University, Kyoto, Japan; 3 Division of Microbiology and Infectious Diseases, Faculty of Dentistry & Graduate School of Medical and Dental sciences, Niigata University, Niigata, Japan; 4 Division of Periodontology, Faculty of Dentistry & Graduate School of Medical and Dental sciences, Niigata University, Niigata, Japan; 5 Faculty of Pharmaceutical Sciences, Tokushima Bunri University, Tokushima, Japan; 6 Department of Chemistry and Functional Molecule, Faculty of Pharmaceutical Sciences, Tokushima Bunri University, Tokushima, Japan; 7 Division of Anatomy and Cell Biology of Hard Tissue, Faculty of Dentistry & Graduate School of Medical and Dental sciences, Niigata University, Niigata, Japan

**Keywords:** Biofilm, Detachment, *Streptococcus*, Functional molecule, Glucosyltransferase, Gene expression

## Abstract

**Background:**

Sulfated vizantin, a recently developed immunostimulant, has also been found to exert antibiofilm properties. It acts not as a bactericide, but as a detachment-promoting agent by reducing the biofilm structural stability. This study aimed to investigate the mechanism underlying this activity and its species specificity using two distinct ex vivo oral biofilm models derived from human saliva.

**Results:**

The biofilm, composed mainly of the genus *Streptococcus* and containing 50 μM of sulfated vizantin, detached significantly from its basal surface with rotation at 500 rpm for only 15 s, even when 0.2% sucrose was supplied. Expression analyses for genes associated with biofilm formation and bacterial adhesion following identification of the *Streptococcus* species, revealed that a variety of *Streptococcus* species in a cariogenic biofilm showed downregulation of genes encoding glucosyltransferases involved in the biosynthesis of water-soluble glucan. The expression of some genes encoding surface proteins was also downregulated. Of the two quorum sensing systems involved in the genus *Streptococcus*, the expression of *luxS* in three species, *Streptococcus oralis*, *Streptococcus gordonii*, and *Streptococcus mutans*, was significantly downregulated in the presence of 50 μM sulfated vizantin. Biofilm detachment may be facilitated by the reduced structural stability due to these modulations. As a non-specific reaction, 50 μM sulfated vizantin decreased cell surface hydrophobicity by binding to the cell surface, resulting in reduced bacterial adherence.

**Conclusion:**

Sulfated vizantin may be a candidate for a new antibiofilm strategy targeting the biofilm matrix while preserving the resident microflora.

**Supplementary Information:**

The online version contains supplementary material available at 10.1186/s12866-020-02033-w.

## Background

Numerous and diverse microorganisms reside in the intraoral environment, creating multispecies microbial communities that form an oral biofilm [1]. Within such a biofilm population, cells demonstrate genetic and physiological heterogeneity as they adapt to the local environmental conditions [2]. Unless these biofilms are appropriately controlled, they accumulate and contribute to tooth decalcification and periodontal inflammation [3–5].

In contrast, the natural oral microbiota has a symbiotic or mutualistic relationship with the host, and delivers important benefits. The commensal bacteria act as developers of proper tissue structure and function as well as host protection simply by niche occupation [6]. Thus, the ideal antibiofilm strategy is to control oral biofilms to levels compatible with oral health while preserving the natural and beneficial properties of the resident oral microflora [7].

Antimicrobial agents have been formulated into many oral care products to augment mechanical elimination [8, 9]. The mechanisms of most of these agents are based on a bactericidal effect by retaining them in the mouth for relatively long periods at sublethal levels. However, increasing evidence indicates some adverse influences of an antibiofilm strategy relying on bactericidal activity. It has been reported that no or little biofilm structure is removed when oral biofilms are treated with chemical compounds such as mouthwashes [10–12]. The residual structure thus serves as a scaffold for biofilm redevelopment [13]. More recent investigations raised an alarm on the long-term use of antimicrobials due to the risk of the emergence of antimicrobial resistant bacteria [14, 15]. Disruption of this community may create dysbiosis, contributing to dental caries, periodontal disease, and an associated increased risk of various diseases such as diabetes, atherosclerotic vascular diseases, and rheumatoid arthritis [6]. Therefore, an ideal antibiofilm strategy would be to reduce the amount of biofilm quantitatively while maintaining the proportion of each resident bacterial species qualitatively, using a method other than eradication.

Vizantin, which is a derivative of trehalose-6,6-dimycolate, is extracted from *Mycobacterium tuberculosis* and activates the innate immune response through specific binding to the toll-like receptor 4/myeloid differentiation factor 2 protein complex without inducing tumor necrosis factor-α production [16]. Recently, we found that sulfated vizantin (Viz-S), which is a solubilized vizantin, also disrupts biofilms formed by *Streptococcus mutans* [17]. Viz-S inhibits bacterial adhesion without affecting bacterial growth and alters its internal architecture. Although Viz-S does not inhibit biofilm maturation of *S. mutans*, it reduces its structural stability, resulting in biofilm detachment. However, the mechanism associated with this activity and its species specificity remains unclear. This study aimed to answer these questions by utilizing two distinct ex vivo oral biofilm models derived from human saliva.

## Results

### Influence of Viz-S on cytotoxicity and bacterial growth

**Fig. 1:**
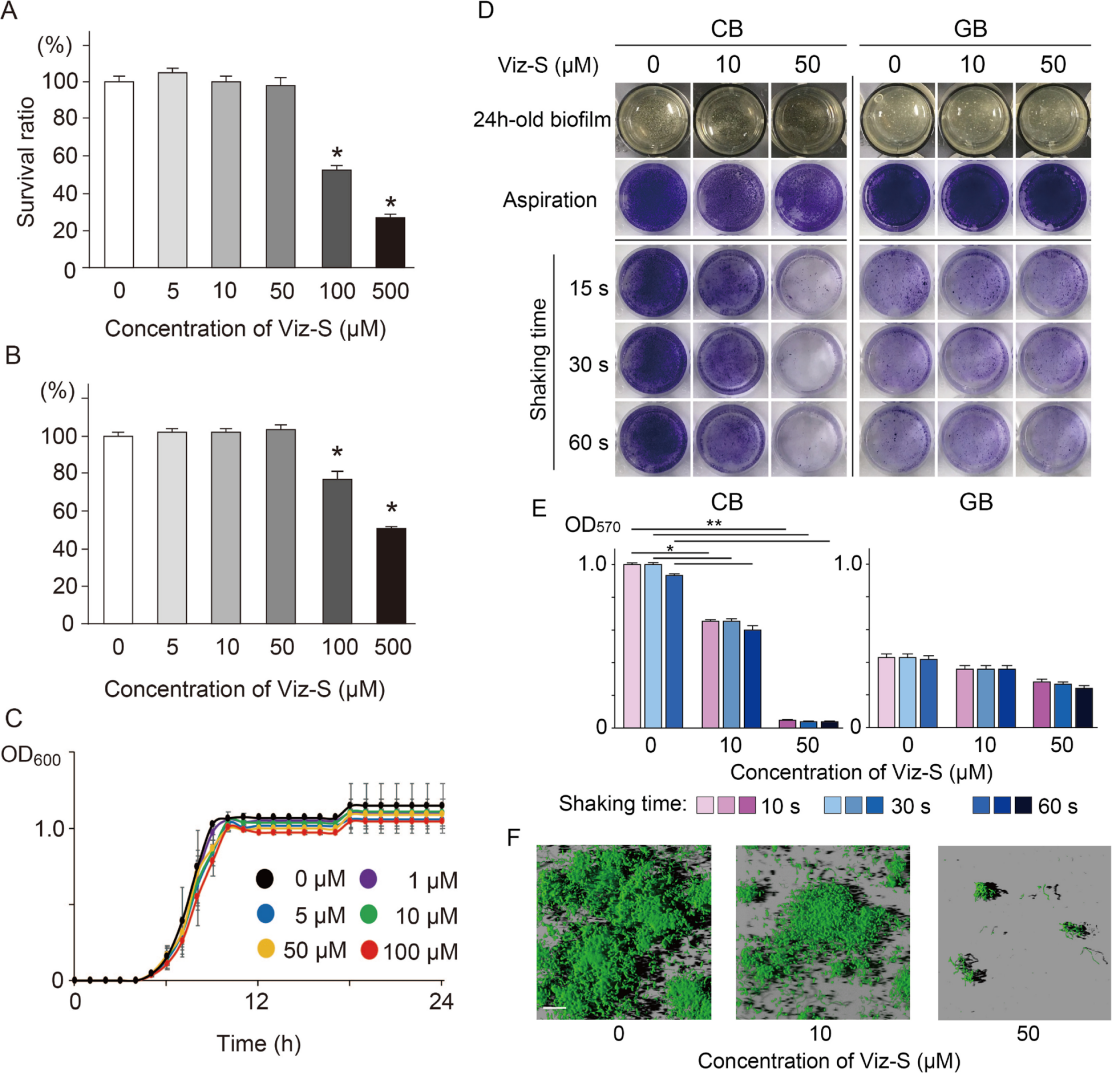
Cytotoxicity of Viz-S in (**a**) human gingival epithelial cells (HGECs) and (**b**) human gingival fibroblasts (HGFs). Cells were incubated in MEM or DMEM containing Viz-S for 3 h at 37 °C. The cellular viability was assessed using an MTT assay (*n* = 6). *p < 0.01, as compared with the control group. **c** Growth curves of bacteria derived from human saliva in the presence or absence of Viz-S (*n* = 4). There were no significant growth differences among the concentrations of Viz-S (*p >* 0.05). **d** Residual biovolumes following shaking motion for 15, 30 and 60 s (*n* = 5). The remaining structure was stained with 0.1% crystal violet. A 24h-old biofilm: Photographs of the biofilm structure after a 24-h incubation. Aspiration: The structure after the supernatant was removed without a washing procedure. **e** Total biomass determined by measuring absorbance at 570 nm (*n =* 5). **p < 0.01, *p < 0.05, compared with the control group. **f** Three-dimensional reconstruction images of residual CB stained with a fluorescent bacterial viability kit following shaking motion for 15 s (*n* = 3). Live bacteria appear fluorescent green (SYTO9) and dead bacteria appear fluorescent red (PI). Scale bar = 30 μm

The cytotoxicity assays showed that Viz-S at concentrations less than 50 μM did not affect the human gingival epithelial cells (HGECs) and human gingival fibroblasts (HGFs) (Fig. 1a and b). When estimating the influence of Viz-S against bacterial growth based on their values of optical density of the culture at 600 nm (OD_600_), no discernible effect was observed. Even at the maximum concentration of 100 μM, Viz-S did not show an antimicrobial effect against bacteria derived from human saliva (Fig. 1c). Considering biological safety as well as protection of resident microflora, Viz-S at a maximum concentration of 50 μM was selected for further analyses.

### Influence of Viz-S on biofilm formation and structural stability

The presence of Viz-S in samples with up to 50 μM of Viz-S did not inhibit maturation in both cariogenic biofilm (CB) and gingivitis biofilm (GB) after 24 h (Table 1). Although the amounts of protein and carbohydrate constituents showed a tendency to increase in a concentration-dependent, there were no significant differences in protein and carbohydrate volumes (*p* > 0.05). CB was relatively richer in the amounts of protein and carbohydrate than GB irrespective of Viz-S.

**Table 1:** Quantitative analysis of the protein and carbohydrate compositions

Concentration of Viz-S	CB		GB	
	Protein	Carbohydrate	Protein	Carbohydrate
0	336.4 ± 6.2	48.7 ± 2.1	169.9 ± 16.0	27.4 ± 2.9
10	364.7 ± 8.4	54.7 ± 1.9	165.3 ± 25.0	28.1 ± 2.5
50	382.7 ± 8.1	58.9 ± 2.5	192.9 ± 31.2	30.5 ± 0.9

Values are presented as mean ± SEM (μg) per well of five replicates. There was no significant difference between the control group and experimental group (*p* > 0.05)

The adherence of CB containing 50 μM Viz-S was not strong and the biofilm was easily peeled off by slight movement (See the supplemental movie file). Viz-S at a concentration of 100 μM prevented the bacteria from bacterial adhesion (See the supplemental movie file). When a shaking motion was applied to CB for only 15 s, the biofilm developed in the presence of 50 μM Viz-S lost its shape, and was almost completely detached from the lower surface (Fig. 1d, e, *p <* 0.05). The GB had a reduced volume after 15-s rotation even in the absence of Viz-S (Fig. 1d, e). CB was structurally more stable than GB if Viz-S was not included. Prolonged agitation did not contribute to the structural degradation of CB in the control (without Viz-S) and in the 10 μM Viz-S group. Three-dimensional reconstruction images also showed that the residual structure of CB containing 50 μM of Viz-S was scarcely detected; however, the remaining bacteria were still alive, showing that all cells were stained with green fluorescence (Fig. 1f). Based on these findings, further analyses were focused on the constituent bacteria in the CB for elucidating the mechanisms associated with this activity.

### Differentiating composition of cariogenic and gingivitis biofilms

**Fig. 2:**
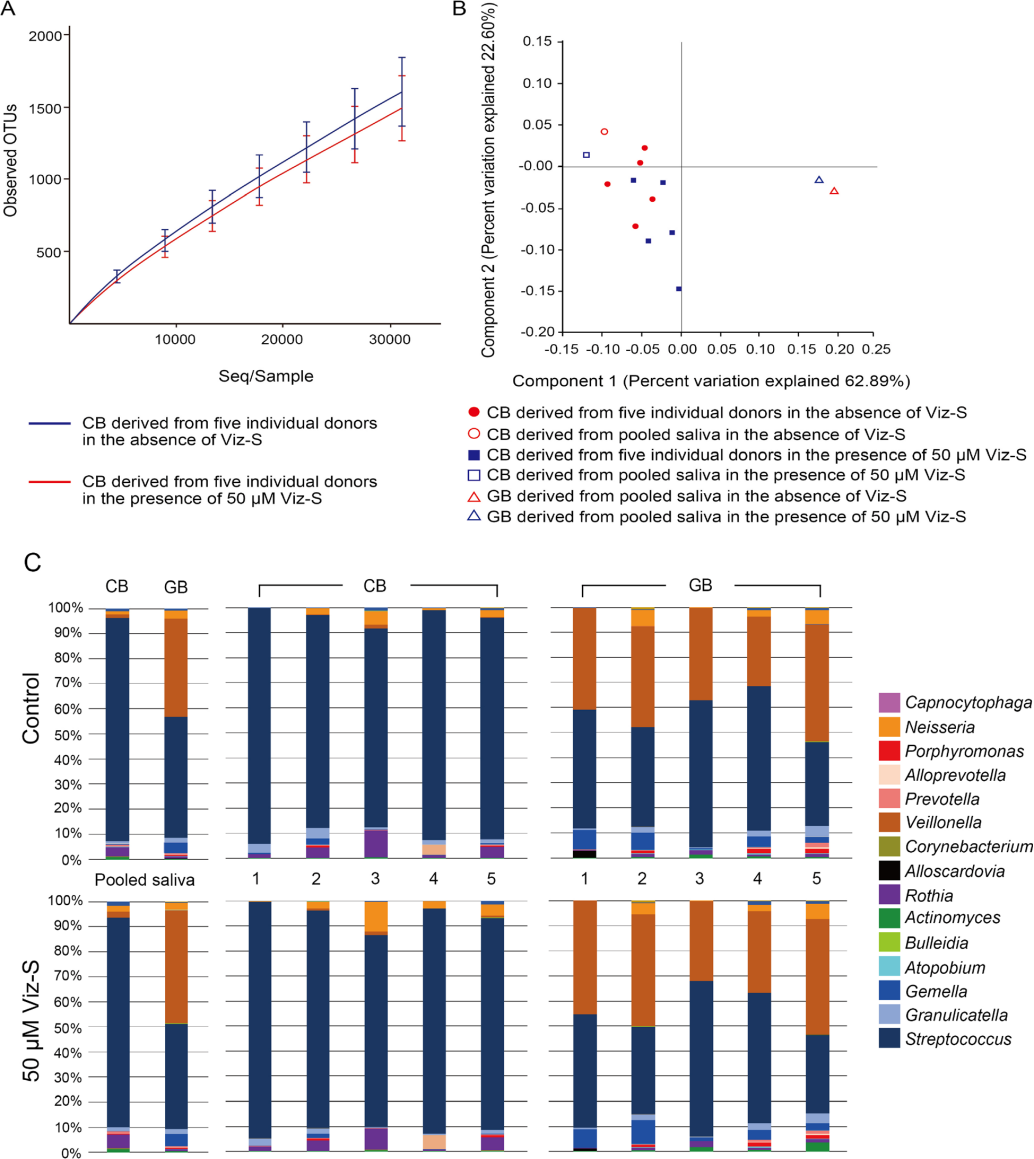
**a** Microbial richness in CB and GB in the absence and presence of 50 μM Viz-S (*n =* 5). **b** Principal component analysis plot of CB and GB. **c** Relative abundance of major bacterial genera. Sequence data for determining the genera in the CB and GB were obtained from 5 individual donors and the pooled saliva from these 5 donors

The microbial compositions of the human saliva biofilms cultured with different nutrient sources, were analyzed by 16S rRNA sequencing. Firmicutes was the most prevalent phylum among all groups constituting 92% of CB in the control group, 88% of CB in the 50 μM group, 93% of GB in the control group, and 94% of GB in the 50 μM group (*p* > 0.05). At the genus level, 69 genera in total were detected and 15 genera of these existed at a rate of > 0.1%. 16S sequencing of CBs and GBs revealed no significant differences in their richness (Fig. 2a). The Shannon diversity index of the CB in the control group (2.67 ± 0.22) and CB in the 50 μM group (2.63 ± 0.15) were significantly lower than those of the GB in the control group (3.52 ± 0.35) and GB in the 50 μM group (3.59 ± 0.34) (*p <* 0.05), indicating that the composition of the CB was less diverse than that of the GB. However, the addition of Viz-S did not influence the diversity of the biofilm (*p* > 0.05). Principal component analysis showed that individual CBs were clearly separated from GB derived from pooled saliva with or without Viz-S (Fig. 2b). Beta diversity did not differ between the CB in the control group and CB in the 50 μM group (*p* = 0.33). LefSe analysis also showed no significant differences in the OTUs at the genus level between the control and 50 μM Viz-S groups. Principal component analysis of individual GBs is shown in Fig. S1.

At the genus level, the most abundant OTU in the CB derived from the pooled saliva (control) was *Streptococcus* (89.1%; 29,882 reads), followed by *Rothia* (3.9%; 1308 reads) (Fig. 2c). Similarly, the CB with 50 μM Viz-S presented *Streptococcus* (83.6%; 31,862 reads) as the most abundant followed by *Rothia* (5.5%; 2096 reads). The *Streptococcus* and *Veillonella* genera were the most dominant in the GB derived from the pooled saliva (control), each accounting for 48.5% (11,578 reads) and 39.0% (9310 reads) of the sequences, respectively. GB in the presence of 50 μM Viz-S revealed that *Streptococcus* (42.0%; 10,385 reads) was the most abundant, followed by *Veillonella* (45.4%; 11,226 reads).

q-PCR analysis of bacterial strains in the CB for the control group showed that *Streptococcus salivarius* was the most abundant species (30%) followed by *S. oralis* (18%), *S. sanguinis* (18%), *S. gordonii* (10%), *S. mitis* (8%), and *S. mutans* (2%). The bacterial flora in the CB developed in the presence of 50 μM Viz-S had a similar bacterial composition as those in the CB control, revealing 28% for *S. salivarius*, followed by *S. oralis* (18%), *S. sanguinis* (16%), *S. gordonii* (12%), *S. mitis* (10%), and *S. mutans* (2%). *S. sobrinus* was not detected from the pooled saliva sample as well as the CBs.

q-PCR analysis of bacterial strains in the GB for the control group showed that *S. sanguinis* was the most abundant species (25%), followed by *S. salivarius* (22%), *S. oralis* (12%), *S. mitis* (12%), and *S. gordonii* (8%). The bacterial flora in the GB developed in the presence of 50 μM Viz-S had a similar bacterial composition as those in the GB control, revealing 23% for *S. sanguinis*, followed by *S. salivarius* (18%), *S. oralis* (16%), *S. mitis* (12%), and *S. gordonii* (10%). *S. mutans* and *S. sobrinus* were not detected from both the control and 50 μM Viz-S group.

To clarify the mechanism for enhanced disruption in the CB containing 50 μM of Viz-S, further analyses were focused on gene expression changes in the genus *Streptococcus*.

### Expression analysis of genes associated with biofilm formation in CB and GB

**Fig. 3:**
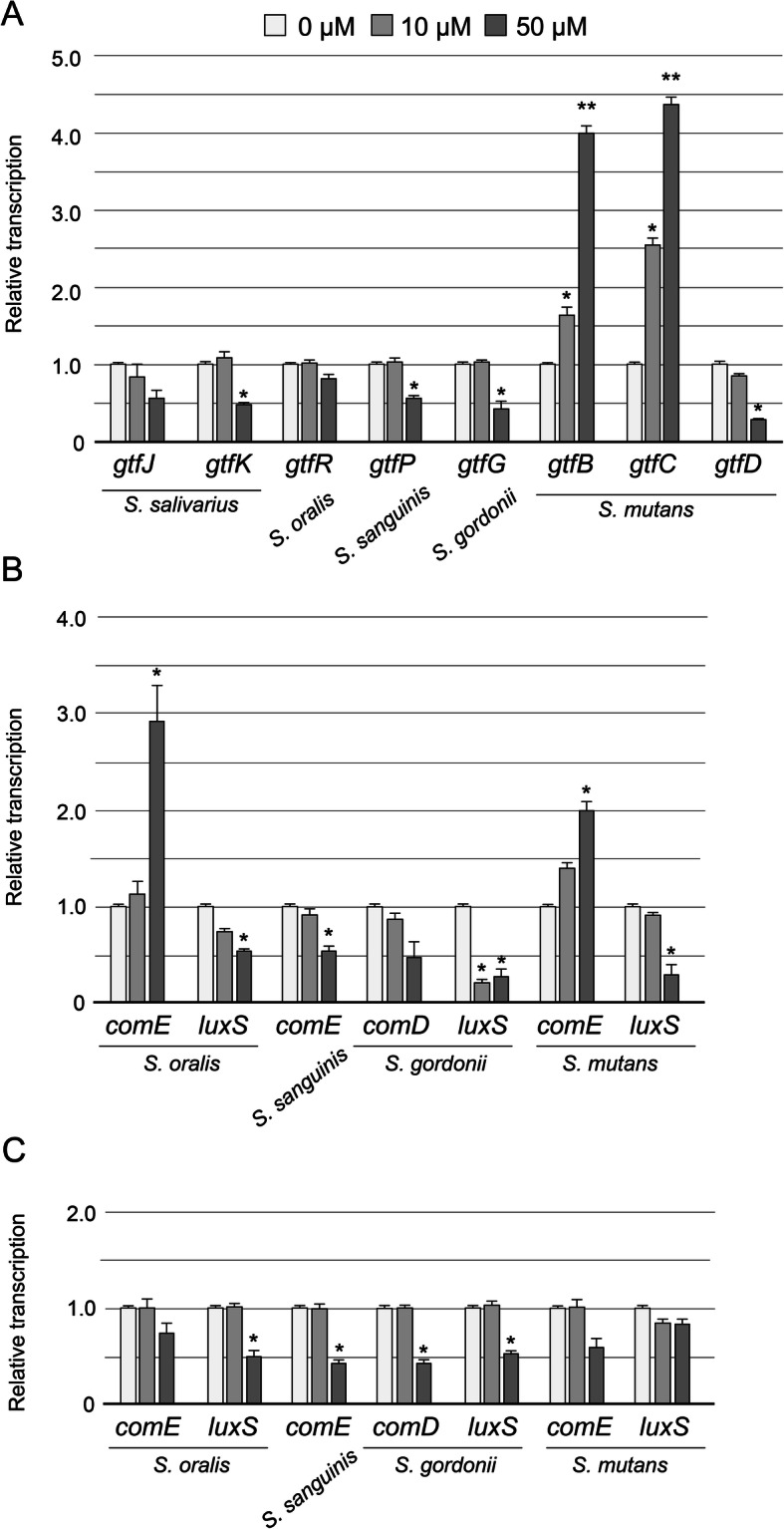
Expression profiles of genes associated with biofilm formation in CB and GB (*n* = 6). **a** *gtf* genes in CB, genes related to QS in CB (**b**) and GB (**c**). *p < 0.05, **p < 0.01, compared with the control group

The expression profiles of fifteen genes in genus *Streptococcus* assumed to participate in the formation of CB and GB after 24 h incubation are summarized in Fig. 3. In the CB containing 50 μM Viz-S, the transcription of several glucosyltransferase (*gtf*) genes involved in the biosynthesis of water-soluble glucan, including *gtfK* for *S. salivarius*, *gtfP* for *S. sanguinis*, *gtfG* for *S. gordonii*, and *gtfD* for *S. mutans*, were significantly downregulated (Fig. 3a, *p <* 0.05). In contrast, the transcription of *gtfB* and *gtfC*, associated with the synthesis of water-insoluble glucans in *S. mutans*, was significantly upregulated compared to those in the control group (*p <* 0.01). There were also significant differences between the control and the 10 μM group in the transcription of *gtfB* and *gtfC* (*p <* 0.05).

The expression of genes involved in the quorum sensing (QS) system, such as those encoding competence stimulating peptide (CSP)-ComDE [18] and LuxS [19], was also examined. The expression of *luxS* for three species in the 50 μM group was significantly downregulated compared with those in the control group (Fig. 3b, *p <* 0.05). *comD* and *comE* expression were found to depend on the bacterial species. The expression of *comE* for both *S. oralis* and *S. mutans* in the 50 μM Viz-S group was significantly upregulated compared to that in the control group, whereas *comE* for *S. sanguinis* was significantly downregulated (Fig. 3b, *p <* 0.05). The expression of *comD* for *S. gordonii* was not significantly changed in the presence of Viz-S.

Gene transcription in the GB containing 50 μM Viz-S was similar to that of CB in the 50 μM group, except *comE* for *S. oralis* and for *S. mutans*. All gene transcripts were downregulated or unchanged (Fig. 3c).

### Expression analysis of genes associated with bacterial adhesion in streptococci

**Fig. 4:**
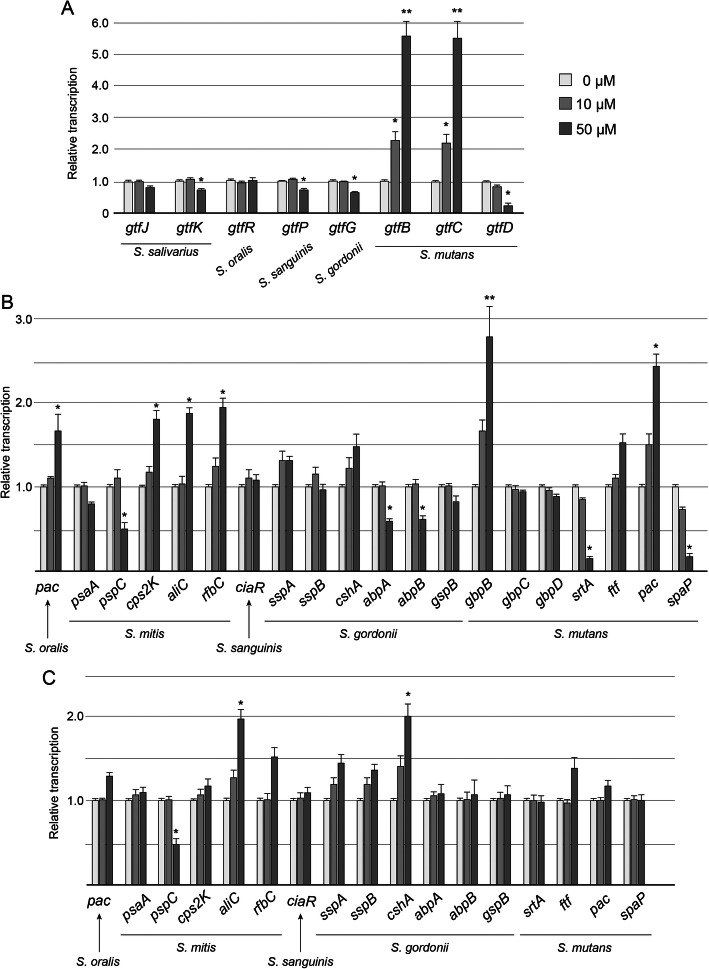
Expression profiles of genes associated with bacterial adhesion (*n =* 6). Transcription of *gtfs* (**a**) and genes related to surface antigens (**b**) in the genus *Streptococcus* when the saliva mixture was incubated in 1/4 strength BHI broth with 0.2% sucrose. **c** Transcription of genes related to surface antigen in the genus *Streptococcus* when the saliva mixture was incubated in 1/4 strength BHI broth with 10% FBS. *p < 0.05, **p < 0.01, compared with the control group

The expression profile of 28 genes assumed to participate in bacterial adherence following incubation for 4 h in the genus *Streptococcus,* is summarized in Fig. 4. When the saliva mixture was incubated in 1/4 strength BHI broth with 0.2% sucrose containing 50 μM of Viz-S, the transcription of several *gtf* genes involved in the biosynthesis of water-soluble glucan, including *gtfK* in *S. salivarius*, *gtfP* in *S. sanguinis*, *gtfG* in *S. gordonii*, and *gtfD* in *S. mutans*, was significantly downregulated (Fig. 4a, *p <* 0.05). In contrast, the transcription of *gtfB* and *gtfC* in *S. mutans* was significantly upregulated compared to that in the control group (*p <* 0.01). The expression of genes that encode surface antigens varied among the bacterial species (Fig. 4b). Transcription of *pspC* in *S. mitis*, *abpA* and *abpB* in *S. gordonii*, and *srtA* and *spaP* in *S. mutans* in the 50 μM Viz-S group was also significantly downregulated compared with that in the control group (*p* < 0.05). In contrast, the transcription of both *pac* in *S. mutans* and a gene encoding the PAc protein homolog [20] in *S. oralis* were significantly upregulated when 50 μM of Viz-S was included in the media. The transcription of *cps2K, aliC,* and *rfbC* in *S. mitis* and glucan-binding protein B (*gbpB*) in *S. mutans* in the 50 μM Viz-S group were also significantly upregulated compared with those in the control group.

When the saliva mixture was incubated in 1/4 strength BHI broth with 10% FBS containing 50 μM of Viz-S, the transcription of most genes remained unchanged except for *pspC* and *aliC* in *S. mitis* and *cshA* in *S. gordonii* (Fig. 4c).

### Binding of vizantin on bacteria and altered hydrophobic property

**Fig. 5:**
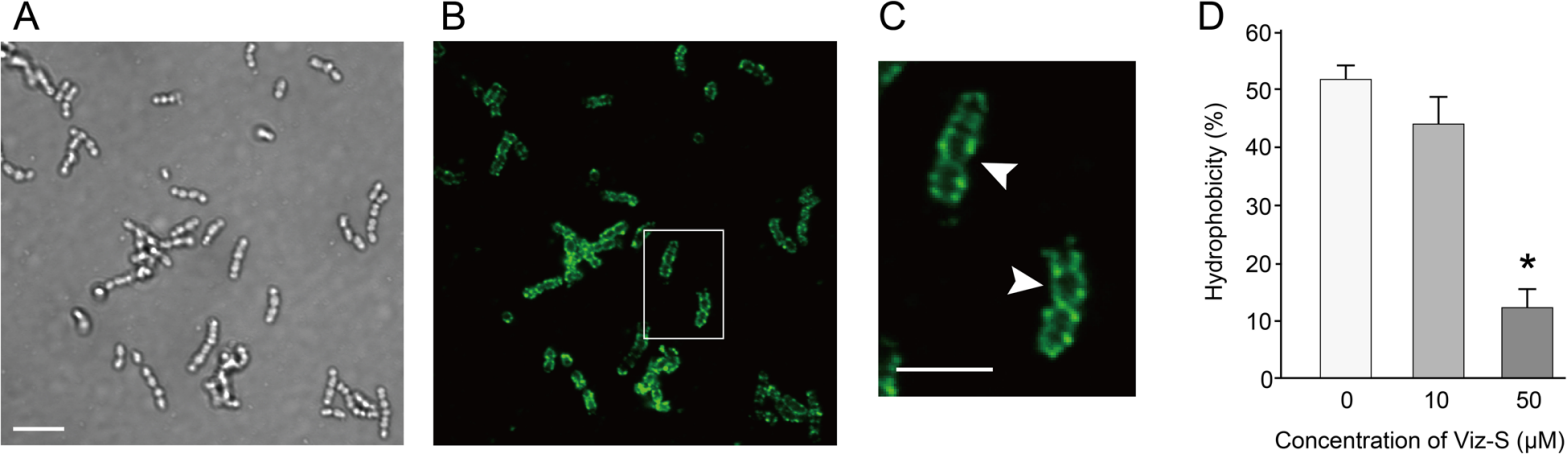
**a**-**c** Representative image of localization of Bodipy-labelled vizantin (Fluo-Viz) on the cell surface. **a** Transmission image of glass bottom surface. Almost all adhered cells were cocci (An additional zoom of 3× using a 100× oil-immersion objective lens). Scale bar = 5 μm. **b** Fluorescence image. Fluo-Viz (green) bound to the bacterial surface. **c** Higher magnification of the area indicated by the squares in B (an additional zoom of 5×). Arrowheads show the chains of Streptococci. Scale bar = 5 μm. **d** Hydrophobic property determined using microbial adhesion to hydrocarbon (MATH) test following exposure to 10 or 50 μM of Viz-S for 10 min (*n =* 6). *p < 0.05, compared with the control

Almost all successfully attached bacteria on the glass bottom during first two hours were cocci (Fig. 5a). Fluo-Viz bound to the cell surface of the bacteria, demonstrating chains of *Streptococci* (Fig. 5b, c). The microbial adhesion to hydrocarbon (MATH) test showed that Viz-S interacted with the surfaces of the microorganisms in saliva and rendered them more hydrophilic. Cells treated with the 50 μM concentration of Viz-S showed that hydrophobicity was reduced to one-fourth in the untreated cells (*p* < 0.05, Fig. 5d).

### Bacterial adhesion to a hydroxyapatite disc following exposure to Viz-S

The number of microorganisms adhering to the hydroxyapatite surface in the bacterial adhesion assay were decreased significantly when the bacteria were exposed to 50 μM of Viz-S for 10 min. The mean number (Mean ± SEM) of viable microorganisms that had attached in 20 min was 4.49 ± 0.05 for the control and 3.70 ± 0.09 for the 50 μM group, showing a significant difference between the control and 50 μM groups (*p <* 0.05).

## Discussion

In this study, we demonstrated that Viz-S at a concentration of 50 μM caused detachment of the biofilm composed mainly of the genus *Streptococcus* despite supplementation with sucrose as a nutrient source. We found that 50 μM of Viz-S did not affect the bacterial growth and viability; additionally, microbiome analysis revealed that 50 μM of Viz-S did not alter the bacterial composition in CB under the culture conditions used in this study. In developing antibiofilm strategy using a chemical compound, preserving a balanced microbiome is one of the critical factors to be considered. Disruption of equilibrium in the oral ecosystem creates a dysbiosis either by overgrowth of specific microorganisms or by changes in the local host response where the community can support a disease state [6, 21].

Viz-S at less than 50 μM did not demonstrate cytotoxicity in HGECs and HGFs (Fig. 1a and b). We also performed a cytokine assay to determine whether Viz-S stimulated the production of inflammatory cytokines in HGECs and human monocyte/macrophage (THP-1) cells. The results showed that the levels of interleukin-1 beta (IL-1β), tumor necrosis factor (TNF), and interleukin-8 (IL-8) in both HGECs (data not shown) and THP-1 following 50 μM of Viz-S treatment did not increase (Fig. S2). These results indicate that Viz-S may be a candidate detachment-promoting agent against early colonizing bacteria (mainly *Streptococcus*) while preserving resident microflora.

The influence of Viz-S on the GB remains unclear because GB showed a reduced volume after 15-s agitation regardless of Viz-S (Fig. 1d). *Veillonella*, the second most abundant genus in GB, act as bridging species to support the colonization and growth of later colonizers including *Porphyromonas gingivalis* rather than in biofilm formation [22]. Many kinds of *Veillonella* species such as *V. denticariosi*, *V. parvula*, *V. rogosae*, *V. atypica*, *V. dispar*, and *V. tobetsuensis* were detected in GB (Fig. S3).

Some agents or molecules have been reported to enable detachment or degradation of oral biofilms [23–26]. However, some of these candidate substances target a specific species or substance. Viz-S seemed to be effective at least for the genus *Streptococcus*.

Considering that the biofilm composed mainly of the genus *Streptococcus* was detached by 50 μM Viz-S in the presence of sucrose, we hypothesized that Viz-S caused detachment of the biofilm through modulation of the sucrose-dependent metabolic activities in *Streptococcus* species. Gene expression analysis in CB showed downregulation of *gtfs* involved in the biosynthesis of water-soluble glucan in the genus *Streptococcus* (Fig. 3a, 4a). Downregulation of water-soluble glucan may weaken cohesion among bacteria. Water-soluble glucan is also reported to play an important role in biofilm formation. Yoshida et al. demonstrated that *gtfP*-deficient *S. sanguinis* exhibits a marked reduction in the amount of water-soluble glucans in the culture supernatant, and produces decreased amounts of extracellular polymeric substances compared to the wild-type [27]. *S. gordonii*, an important pioneer colonizer, has a *gtfG* that synthesizes both α1.3- and α1.6-linked glucans [28]. GTFG is reported to influence biofilm interactions with other bacterial species such as *Candida albicans* [29]. In *S. mutans*, the expression of *gtfD*, which synthesizes water-soluble glucans, was significantly downregulated in the 50 μM group. However, the expression of *gtfB* and *gtfC* was significantly upregulated by more than 4.0-fold (Fig. 3a, 4a). Previous studies have reported that the presence of all three Gtfs at an optimal ratio is necessary for the biofilm stability [30, 31]. For example, Ooshima et al. reported that the highest level of sucrose-dependent adherence was attained when GtfB, GtfC, and GtfD were present at a ratio of 20:1:4 [31]. Matsumoto et al. reported that binding of the soluble glucan synthesized by GtfD to GbpC protein enhanced the sucrose-dependent adhesion [32].

Of the two QS systems in the genus *Streptococcus*, the expression of *luxS* in *S. oralis*, *S. gordonii*, and *S. mutans* was significantly downregulated in the 50 μM Viz-S groups (Fig. 3b). The pathway of autoinducer-2 (AI-2) synthesized by *luxS* can control the expression of genes involved in a variety of metabolic pathways and pathogenic mechanisms. The phenomenon of significant biofilm detachment from the basal surface can be explained to some extent. McNab et al. demonstrated that the inactivation of *S. gordonii luxS* downregulated the expression of *gtfG* [33]. Cuadra-Saenz et al. reported that a dual-species *luxS* mutant biofilm by *S. oralis* and *S. gordonii* had a bigger volume and sparser architecture compared with that formed by the wild-type pair [34]. Yoshida et al. reported that biofilm formation by the *luxS* mutant of *S. mutans* in 0.5% sucrose defined medium was markedly attenuated compared to that of the wild type, and the *gtfB* and *gtfC* genes, but not the *gtfD* gene, were upregulated in the mid-log growth phase [19]. In addition, the *luxS* mutant exclusively formed very large clumps compared to the biofilm formed by the wild type and its structure was easily detached by washing. Further, the hydrophobicity of the *luxS* mutant strain was lower than that of the wild-type strain [35], suggesting that the adhesion property was likely to decrease. From these findings, downregulation of *luxS* expression may alter the biofilm structure that tends to be dispersed easily.

Oral streptococci express multiple adhesins that enable them to adhere to human teeth and aggregate with each other. Among a limited number of adherence-associated genes analyzed in this study, only five of twenty genes were significantly downregulated (Fig. 4b). The downregulation of these genes may influence bacterial adhesion. For example, *S. gordonii* produces two amylase binding proteins: amylase binding protein A (AbpA) and AbpB. AbpA acts as the major receptor of salivary amylase binding to the bacterial surface [36]. AbpA-deficient strains show decreased adherence to amylase-coated hydroxyapatite [37]. Downregulation of *abpA* and *abpB* may thus contribute to the attenuated adhesion. In *S. mutans* containing 50 μM Viz-S, the expression of *srtA* (sortase A) and *spaP* (surface protein), which are associated with non-sucrose metabolism, were significantly downregulated compared with those in the control group. In contrast, the expression of *pac* (cell surface protein antigen c) was significantly upregulated. PAc is known to participate in bacterial adherence to teeth via interaction with the salivary pellicle [38]. Further, the expression of a gene in *S. oralis* that encodes an amino acid sequence with 76.2% similarity to the PAc protein precursor of *S. mutans* and 73.8% homology with the SpaA protein precursor of *S. sobrinus* [20]*,* was significantly upregulated. It is unclear how the expression of these genes contributes to bacterial adhesion. As a variety of microorganism surface proteins are assumed to be involved with bacterial adhesion, the whole mechanism of biofilm detachment caused by Viz-S remains unclear.

To investigate whether Viz-S prevents bacterial attachment non-specifically, MATH test and bacterial adhesion assay were also performed. The bacteria bound to Viz-S showed decreased hydrophobicity (Fig. 5d) and inhibited bacterial adhesion. Thus, Viz-S binding affects bacterial adhesion non-specifically, and may also influence cell-to-cell and cell-to-host interactions.

Although we have found some clues to elucidate the mechanism associated with Viz-S activity, further investigations are needed to demonstrate how the transcriptional changes induced by Viz-S contribute to promote biofilm detachment at the protein level.

## Conclusions

Numerous and diverse microorganisms reside in our intraoral environment, and we all coexist with oral biofilms. Thus, using a chemical antibiofilm strategy in the oral cavity would be different from the approaches used against medical pathogens elsewhere in the body. The aim for chemical control would be to prevent biofilm accumulation rather than eradication, while still preserving the benefits of the normal resident oral microflora.

In this study, Viz-S showed the ability to detach biofilms composed mainly of the genus *Streptococcus* through two possible mechanisms. As a specific reaction, Viz-S affects the expression of genes associated with bacterial adhesion and biofilm formation in *Streptococcus* species. Reduced structural stability might facilitate the detachment of these biofilms. As a non-specific reaction, Viz-S reduces bacterial adherence by changing the surface property of cells, without affecting bacterial growth or causing cytotoxicity. However, these mechanisms remain undetermined, and the efficacy against periodontal pathogens is necessary to elucidate by further analyses.

Within the limitations of this in vitro study, it was demonstrated that Viz-S may be a candidate for a new antibiofilm strategy targeting the biofilm matrix while preserving the resident microflora. Further clinical studies are needed to evaluate the impact of Viz-S for the control of oral biofilms.

## Methods

### Cellular toxicity of sulfated vizantin

Viz-S was prepared as described in a previous report [39]. HGECs (Ca9–22; RIKEN BioResource Center, Ibaraki, Japan) and HGFs (ATCC PCS-201-018; Summit Pharmaceuticals International, Tokyo, Japan) were grown to 90% confluence in minimum essential medium (MEM; Life Technologies, Grand Island, NY, USA) or Dulbecco’s modified Eagle’s medium (DMEM; Wako Pure Chemical Industries, Osaka, Japan) containing 10% fetal bovine serum (FBS) (Japan Bio Serum, Hiroshima, Japan) and 1% penicillin-streptomycin (Wako Pure Chemical Industries) at 37 °C in 5% CO_2_. The cells were then seeded at a density of 1 × 10^5^ cells/ml in MEM or DMEM to 96-well plates. The cells were then incubated in the presence of Viz-S at concentrations of 0, 5, 10, 50, 100, and 500 μM for 3 h at 37 °C. Cellular viability was assessed using a methyl thiazolyl tetrazolium (MTT) assay [40], performed with a total of six replicates per treatment.

### Saliva collection

Unstimulated saliva was collected from five healthy volunteers. Informed consent was obtained from all participating subjects under a protocol reviewed and approved by the Niigata University Ethics Committee (25-R16–08-15). The donors abstained from food and drink intake for 2 h prior to saliva donation. The saliva was diluted twofold with sterile 60% glycerol and was stored at − 80 °C until used [41]. The same volume of saliva samples was pooled in a tube immediately before use, and a single pooled sample was used as bacterial inoculation for biofilm preparation [42, 43].

### Growth curve assay

Pooled saliva (40 μl) was added to 2 ml of brain heart infusion (BHI) broth (Difco Laboratories, Detroit, MI, USA) using a 24-well tissue culture plate (Falcon™, Fisher Scientific, Waltham, MA, USA) containing 0, 5, 10, 50, and 100 μM of Viz-S. The plate was statically incubated at 37 °C under anaerobic conditions. The OD_600_ was recorded at hourly intervals. A growth curve assay was performed with a total of four replicates per treatment.

### Detachment property of Viz-S in CB and GB

Two distinct oral biofilms, namely cariogenic and gingivitis biofilms, derived from healthy human saliva, were used as described previously, with a slight modification [42, 43]. The media used were 1/4 strength BHI broth with 0.2% sucrose for the cariogenic biofilm (CB), and 1/4 strength BHI broth with 10% fetal bovine serum for the gingivitis biofilm (GB). A 24-well plate was coated with 10% sterile saliva solution prepared, as described previously [44], for 2 h prior to the incubation (pellicle formation). Then, 40 μl of a single pooled sample from five donors was used to inoculate the two types of media to form biofilms. The CB and GB were developed in a 24-well plate containing Viz-S at concentrations of 0, 10, and 50 μM for 24 h at 37 °C under anaerobic conditions.

After 24 h, the medium in the wells was replaced with phosphate-buffered saline (PBS), without agitation. The biofilms were rotated at 500 rpm for 15, 30, and 60 s at 37 °C using a Thermo-Shaker (MyBL-P25, AS ONE, Osaka, Japan). The residual biofilms were stained with 0.1% crystal violet (CV) for 15 min. The wells were washed three times with PBS to remove the unbound CV and dried for 2 h at 37 °C. The bound CV was collected by adding 1 ml of 30% (v/v) acetic acid for 5 min, and the total biomass was determined by measuring the absorbance at 570 nm with a colorimeter (CO7500®, Funakoshi Co. Ltd., Tokyo, Japan) [17].

Morphological observation of the remaining structure in the CB group following a shaking motion for 15 s was performed using confocal laser scanning microscopy analysis. The residual structure was stained with a fluorescent bacterial viability kit (LIVE/DEAD® BacLight™ Bacterial Viability Kit) (Thermo Fisher Scientific, Waltham, MA, USA) for 30 min at room temperature in the dark. The biofilm was imaged using a confocal laser scanning microscope with Ar 488-nm and He-Ne 543-nm lasers (CLSM; FluoView™ 300, Olympus, Tokyo, Japan). The filters were set to 510–530 nm for detection of the SYTO 9 stain, and to > 610 nm for propidium iodide (PI). A water-immersion objective lens (× 60) was used. Stacks of the fluorescence images were collected every 0.45 μm in the Z dimension, and three-dimensional reconstruction was carried out using Imaris® software (Bitplane AG, Zurich, Switzerland) [17]. This assay was performed with a total of three replicates per treatment.

### Quantitative analysis of protein and carbohydrate composition

CB and GB were developed in the absence and presence of Viz-S for 24 h, as described previously, in a 6-well plate. The supernatant was aspirated and the biofilm was washed twice except for CB in the 50 μM Viz-S group, as the biofilm developed in the presence of 50 μM Viz-S detached readily from the surface by a washing step. The biofilms were collected using a cell scraper (TPP Techno Plastic Products AG, Trasadingen, Switzerland). The collected biofilm was washed twice with PBS. The bacterial suspension was mechanically pulverized with Lysing Matrix B (MP Biomedical, Santa Ana, CA, USA) using a MagNA Lyser (Roche Diagnostic GmbH, Penzberg, Germany) at a speed of 7000 rpm for 30 s. The amount of protein in the biofilms was determined using a bicinchoninic acid (BCA) Protein Assay Kit (T9300A; Takara Bio Inc., Shiga, Japan) according to the manufacturer’s instructions. After incubation for 30 min, absorbance at 550 nm was measured using a colorimeter. The amount of carbohydrate in the biofilm was measured as described previously [12] with slight modification. Briefly, 500 μl of a 5% aqueous solution of phenol was added to 500 μl of supernatant, incubated for 20 min, and then 2.5 ml of concentrated sulfuric acid was added. The OD at 490 nm was measured using a colorimeter. This assay was performed with a total of five replicates per treatment.

### Microbiome analysis

To determine the composition of biofilms cultured from the pooled sample in the absence and presence of 50 μM Viz-S, genomic DNA was extracted using a NucleoSpin® Microbial DNA (MACHEREY-NAGEL GmbH & Co. KG, Duren, Germany) according to the manufacturer’s instructions. The bacterial microbiota was investigated by targeted 16S rRNA gene (V3-V4 region) sequencing using the Illumina MiSeq system (2× 300 bp paired-end reads) (Illumina, CA, USA) as described previously with some modification [45] (Bioengineering Lab. Co., Ltd., Kanagawa, Japan). Two-step polymerase chain reaction (PCR) procedures were performed to generate amplicon libraries. Ambiguous bases, low quality reads, and sequences with a read length below 200 bp were discarded. The remaining sequences were clustered into phylotypes using QIIME with a minimum coverage of 99% and a minimum identity of 97%. A representative sequence for each operational taxonomic unit (OTU) was selected for taxonomic assignment with reference to the EzBioCloud 16S database. The protocols for amplicon libraries are summarized in Table S1.

The CB and GB derived from each individual saliva sample were also subjected to 16S rRNA gene sequencing for bacterial diversity analysis. Alpha diversity was evaluated by the number of observed OTUs and the Shannon diversity index. Beta diversity was measured as the weighted UniFrac distance based on the OTU table and the difference between CB in the absence of Viz-S and that in the presence of 50 μM was compared using a PERMANOVA. LEfSe, an algorithm for identifying the genomic taxa whose relative abundances differ significantly between two groups, was used for further analysis by a company (Bioengineering Lab. Co., Ltd.).

### Quantitative polymerase chain reaction (q-PCR) analysis

To determine the species composition of the genus *Streptococcus* in the CB and GB, q-PCR analysis was performed as described previously [46–48]. Briefly, standard curves of representative species were first created. Serial 10-fold dilutions (10^4^–10^9^ cells/ml) of *Streptococcus salivarius* ATCC 27945, *Streptococcus oralis* ATCC 10557, *Streptococcus mitis* ATCC 49456, *S. gordonii* ATCC 10558, *S. mutans* UA159, and *Streptococcus sanguinis* ATCC 10556 in the logarithmic phase were prepared. Each genome DNA was extracted as described above, and q-PCR amplification was performed on the StepOnePlus real-time PCR system (Thermo Fisher Scientific) using the SYBR Green detection protocol according to the manufacturer’s instructions. Species-specific primers for 16S rRNA genes are listed in Table S2. The primer sequences were obtained from those used in previous studies [47, 49] or were newly designed in this study. The genome sequences were retrieved from GenBank, and the real-time PCR primers were designed using Primer-BLAST. The primer specificity was checked using the BLAST tool on the NCBI website, and availability in the pooled saliva sample was confirmed by detecting the amplified product obtained by PCR. Each standard curve was generated by plotting the threshold cycle (C_T_) value against the known bacterial amount. CB and GB developed for 24 h in a six-well plate were collected using a cell scraper, followed by genomic DNA extraction. The relative frequency of *Streptococcus* species was analyzed by q-PCR.

### Expression analysis of genes associated with biofilm formation in CB and GB cells by real-time PCR

To investigate gene transcription associated with biofilm formation in the absence and presence of Viz-S, the CB and GB after 24 h were collected and washed twice with PBS. The bacterial pellet was resuspended in TRI Reagent (Molecular Research Center, Inc., Cincinnati, OH, USA) and chemomechanically pulverized with Lysing Matrix B using a MagNA Lyser at a speed of 7000 rpm for 30 s. RNA isolation was performed using a Direct-zol RNA kit (Zymo Research, Irvine, CA, USA). The RNA was reverse transcribed using SuperScript VILO Master Mix (Thermo Fisher Scientific), and q-PCR with cDNA was performed on the StepOnePlus real-time PCR system using the SYBR Green detection protocol. The 16S rRNA gene was used as an internal control for data normalization. The primers used in this study are listed in Table S3. The sequences of the primers were obtained from those used in previous studies [50–55] or were designed in this study. The primers were validated as previously described. This assay was performed with a total of six replicates per treatment.

### Expression analysis of adherence-associated genes in the CB and GB cells by real-time PCR

To investigate gene transcription associated with bacterial adhesion in the absence and presence of Viz-S, 480 μl of the saliva mixture was inoculated into 24 ml of 1/4 strength BHI broth with 0.2% sucrose or 1/4 strength BHI broth with 10% FBS containing 0, 10, and 50 μM of Viz-S in a centrifuge tube. The content was stirred at a speed of 150 rpm at 37 °C for 4 h under anaerobic conditions. CB and GB cells were collected by centrifugation (8000×*g* for 5 min), washed thrice with PBS, and used for RNA extraction as described above. The RNA was reverse transcribed, and q-PCR was performed. The 16S rRNA gene was used as an internal control for data normalization. The primers used in this study are listed in Table S4. The primer sequences were obtained from previous studies [53–60] or were newly designed in this study. The primers were validated as previously described. This assay was performed with a total of six replicates per treatment.

### Localization of vizantin in bacteria

Bodipy-labeled vizantin (Fluo-Viz) was prepared according to a previously reported procedure for its synthesis [16]. The pooled saliva (40 μl) was inoculated into 2 ml of 1/4 strength BHI broth with 0.2% sucrose using a glass bottom culture dish (MatTek Corp., Ashland, MA, USA) and incubated for 2 h at 37 °C under anaerobic conditions. The adhered cells were washed twice with PBS; then, Fluo-Viz at a concentration of 50 μM in distilled water was added to the dish, incubated for 45 min at 37 °C, washed twice with PBS, and imaged using a 488 nm laser line and a 510–530 nm band pass filter. An additional zoom of 3× or 5× was applied using a 100× oil-immersion objective lens.

### MATH test

The saliva sample was incubated in BHI broth at 37 °C under anaerobic conditions until mid-exponential growth. The bacterial culture was washed twice with PBS, centrifuged, and resuspended in PBS. The optical density of the sample was adjusted to 0.4 at 550 nm (OD1). The bacterial suspension was exposed to 0, 10, and 50 μM of Viz-S with agitation for 10 min at 37 °C. Then, 1 ml of each treatment was mixed with 200 μl of *n*-hexadecane. The mixtures were vortexed for 1 min, and the phases were allowed to separate before the optical density of the aqueous phase was measured again (OD2). Hydrophobicity was calculated using the following equation: MATH (%) = (OD1-OD2)/OD1 × 100 [61]. This assay was performed with a total of six replicates per treatment.

### Bacterial adhesion assay

Hydroxyapatite (HA) discs measuring 6 mm in diameter and 1.5 mm in thickness (Olympus) were mounted in a flow-cell chamber (Convertible Flow Cell CFCAS0003, IBI Scientific, Dubuque, IA, USA). Two specimens were placed at either end so that their fluid flows did not interfere with each other. The chamber was sterilized using ethylene oxide gas for 4 h. The flow-cell system comprised the bacterial suspension, a peristaltic pump, and a carboy for waste. These components were connected with silicone tubing (Fig. S4).

An adjusted saliva solution of 10 ml was pumped into a chamber at a flow rate of 2 ml/min and kept static for 1 h at 37 °C to allow salivary pellicle formation. The saliva was prepared as described above.

The optical density of the bacterial suspension derived from the pooled saliva in the logarithmic phase was adjusted to 0.025 at 600 nm in 1/4 strength BHI broth with 0.2% sucrose. The suspension was exposed to 50 μM of Viz-S with agitation for 10 min at 37 °C, followed by the connection to a flow-cell system, and pumping into the flow cell at a flow rate of 2 ml/min for 20 min. Adhered bacteria were then collected and enumerated. The bacterial suspension without Viz-S exposure served as the control. A total of 10 discs were used per experimental condition.

### Statistical analysis

Statistical analyses were performed using SPSS® 11.0 (SPSS Inc., Chicago, IL, USA) and Excel Statistics 7.0 (Esumi Co., Ltd., Tokyo, Japan). Where applicable, the data are presented as the mean ± standard error of the mean (SEM). Significance was determined using the Kruskal-Wallis test with a post hoc Steel-Dwass test (for the protein and carbohydrate compositions) or a post hoc Dunnett’s test (for the cellular toxicity assay, crystal violet assay, MATH test, and gene expression analyses). Two-way analysis of variance was used to compare the bacterial growth test results. A paired Student’s *t*-test was used to compare the bacterial adhesion test results.

Shannon diversity indices were compared with a one-way analysis of variance. PERMANOVA was performed on beta diversity. *P* values < 0.05 were considered to indicate statistical significance.

## Supplementary Information

**Additional file 1:** CB structures after 24-h incubation containing Viz-S at concentrations of 0, 50 and 100 µM.  

**Additional file 2:** **Fig. S1** Principal component analysis plot of GB and CB.

**Additional file 3:Fig. S2** Production of inflammatory cytokines in THP-1 cells following Viz-S treatment (*n* = 5). *p < 0.01, compared with the control group (LPS).

**Additional file 4:** **Fig. S3** Identification of *Veillonella* species using the polymerase chain reaction.

**Additional file 5:** **Fig. S4 **Flow-cell system used in this study.

**Additional file 6:** **Table S1** Two-step PCR protocol used in this study.

**Additional file 7:** **Table S2 **16S rRNA primers used in this study.

**Additional file 8:** **Table S3 **Primer sequences used for analyzing the genes associated with biofilm formation by real-time PCR.

**Additional file 9:** **Table S4 **Primer sequences used for analyzing the genes associated with bacterial adhesion by real-time PCR.

## Data Availability

All data are presented in the manuscript and supplemental figures. These data are available from the corresponding author upon reasonable request.

## Abbreviations

Viz-S: Sulfated vizantin
HGEC: Human gingival epithelial cell
HGF: Human gingival fibroblast
MEM: Minimum essential medium
DMEM: Dulbecco’s modified Eagle medium
MTT: 3-(4,5-di-methylthiazol-2-yl)-2,5-diphenyltetrazolium bromide, yellow tetrazole
BHI: Brain heart infusion
OD: Optical density
CB: Cariogenic biofilm
GB: Gingivitis biofilm
FBS: Fetal bovine serum
PBS: Phosphate-buffered saline
CV: Crystal violet
CLSM: Confocal laser scanning microscopy
PI: Propidium iodide
BCA: Bicinchoninic acid
OTU: Operational taxonomic unit
MATH: Microbial adhesion to hydrocarbon
