# Supplementary figures and images for: Sulfated vizantin causes detachment of biofilms composed mainly of the genus Streptococcus without affecting bacterial growth and viability
Source: BMC Microbiol. 2020 Nov 25;20:361. doi: 10.1186/s12866-020-02033-w (PMC7687742; doi:10.1186/s12866-020-02033-w)

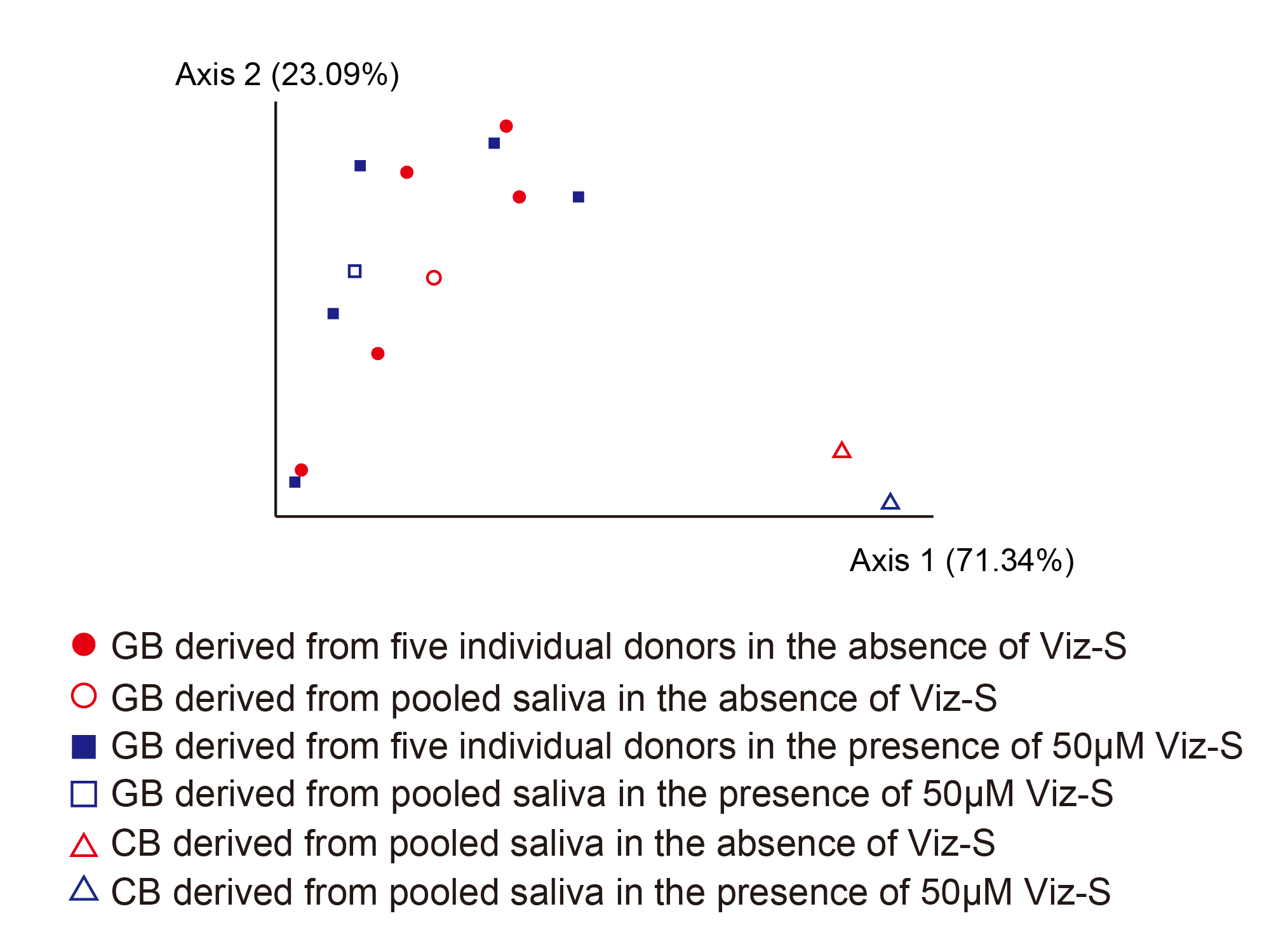

Supplement: Supplementary file 2 — Additional file 2: Fig. S1 Principal component analysis plot of GB and CB. [file 12866_2020_2033_MOESM2_ESM.jpg]

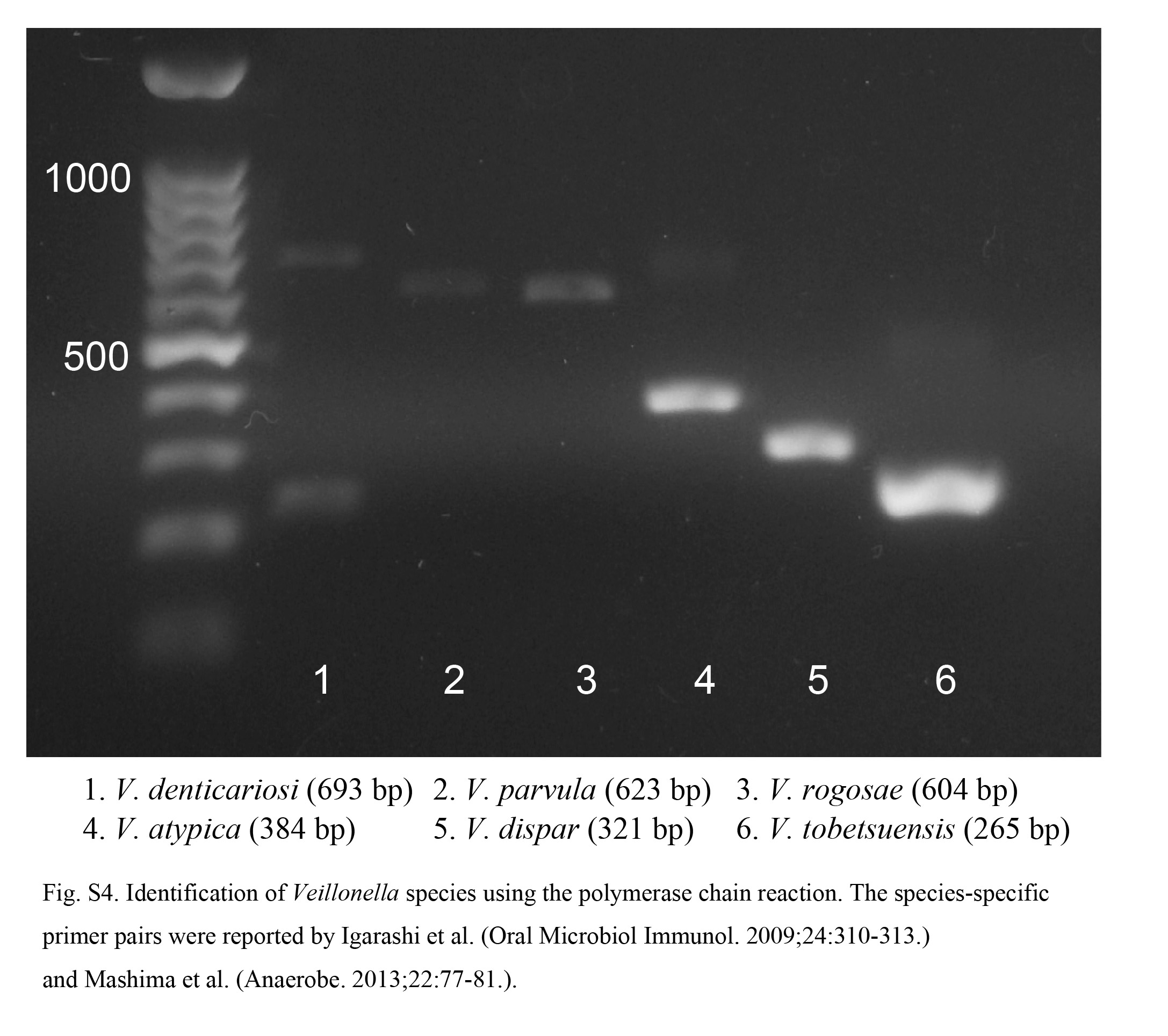

Supplement: Supplementary file 4 — Additional file 4: Fig. S3 Identification of Veillonella species using the polymerase chain reaction. [file 12866_2020_2033_MOESM4_ESM.jpg]

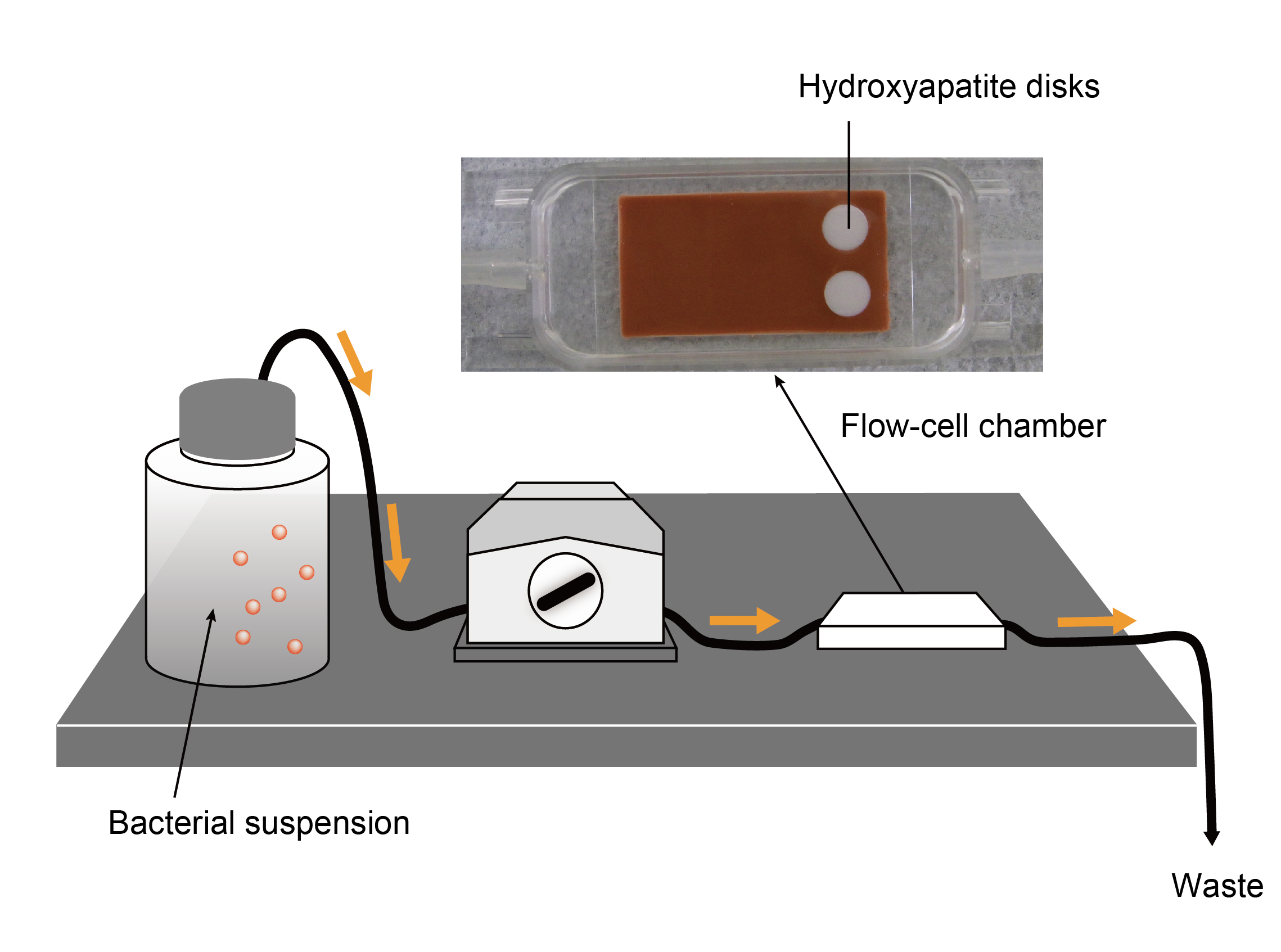

Supplement: Supplementary file 5 — Additional file 5: Fig. S4 Flow-cell system used in this study. [file 12866_2020_2033_MOESM5_ESM.jpg]
