# Supplementary material for: Sulfated vizantin causes detachment of biofilms composed mainly of the genus Streptococcus without affecting bacterial growth and viability
Source: BMC Microbiol. 2020 Nov 25;20:361. doi: 10.1186/s12866-020-02033-w (PMC7687742; doi:10.1186/s12866-020-02033-w)
Supplement: Supplementary file 3 — Additional file 3:Fig. S2 Production of inflammatory cytokines in THP-1 cells following Viz-S treatment (n = 5). *p < 0.01, compared with the control group (LPS). [file 12866_2020_2033_MOESM3_ESM.docx]

**
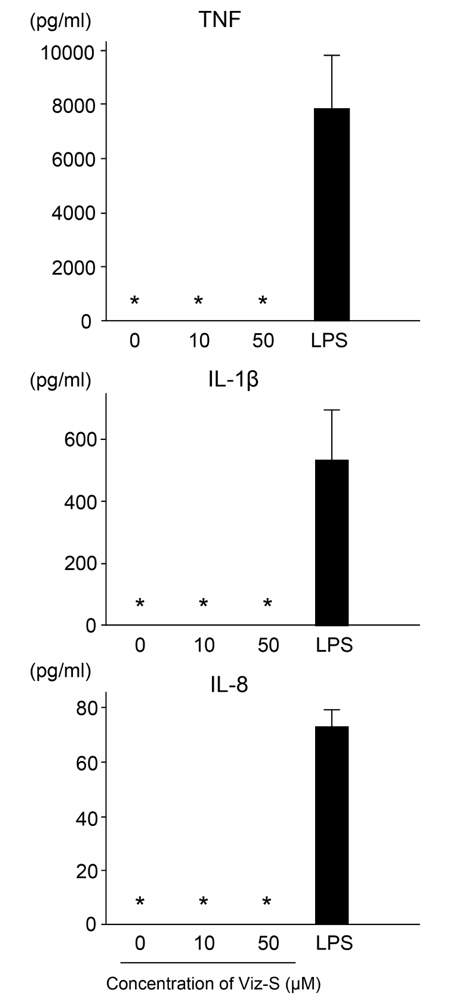
Fig. S3** Production of inflammatory cytokines in THP-1 cells following Viz-S treatment (n = 5). *p< 0.01, compared with the control group (LPS).

Human gingival epithelial cell (HGEC) line Ca9‐22 was grown in minimum essential medium (MEM; WakoPure Chemical Industries, Osaka, Japan) supplemented with 10% FBS (Japan Bio Serum, Hiroshima, Japan), 100 U/mL penicillin and 100 μg/mL streptomycin (Wako Pure Chemical Industries) at 37°C in the presence of 95% air and 5% CO_2_. HGECs were seeded onto 24-well culture plate at a concentration of 5 × 10^5^ cells/mL in the medium followed by treatment with 10 or 50 μM sulfated vizantin. After 1 h, Escherichia coli lipopolysaccharide (LPS) (50 ng/mL; Sigma-Aldrich, St. Louis, MO, USA) was added to the medium followed by incubation for 12 h.

The monocytic cell line THP-1 was maintained in 25 mM HEPES-buffered RPMI 1640, supplemented with 10% fetal bovine serum, 100 U/mL penicillin, and 100 µg/mL streptomycin (Wako Pure Chemical Industries, Osaka, Japan) at 37°C in 95% air and 5% CO_2_. For the experiments, the cells were incubated in a 24-well culture plate at a concentration of 5 × 10^5^ cells/mL in medium supplemented with 200 nM phorbol 12-myristate 13-acetate to induce differentiation into macrophage-like cells. After 48 h incubation, the cells were washed with RPMI 1640 and cultured further in RPMI 1640 for 12 h. Thereafter, cells were treated with 10 or 50 μM sulfated vizantin. After 1 h, 50 ng/mL LPS was added to the medium followed by incubation for 12 h.

The levels of IL-1β, TNF, and IL-8 in the cell culture supernatants were determined by using enzyme-linked immunosorbent assay (ELISA) kits (BioLegend, San Diego, CA, USA).
