# Supplementary material for: Sulfated vizantin causes detachment of biofilms composed mainly of the genus Streptococcus without affecting bacterial growth and viability
Source: BMC Microbiol. 2020 Nov 25;20:361. doi: 10.1186/s12866-020-02033-w (PMC7687742; doi:10.1186/s12866-020-02033-w)
Supplement: Supplementary file 6 — Additional file 6: Table S1 Two-step PCR protocol used in this study. [file 12866_2020_2033_MOESM6_ESM.docx]

**Table S1.** Two-step PCR protocol used in this study.

|  | Reaction mixtures | Amplification reaction condition | Sequence (5'→3') |
| --- | --- | --- | --- |
| 1st PCR | 1µl of 10x Ex Buffer, 0.8µl of 2.5mM dNTPs, 0.5µl of 10µM forward and reverse primers, 2µl of the template DNA (0.5ng/µl), 0.1µl of ExTaq HS*1, 5.1µl of nuclease free water | 2 min denaturation step at 94ºC, followed by 25 cycles of 30 s at 94ºC, 55ºC for 1 min, and a final extension at 72ºC for 5 min | 341F: ACACTCTTTCCCTACACGACGCTCTTCCGATCT- NNNNN-CCTACGGGNGGCWGCAG  805R:  GTGACTGGAGTTCAGACGTGTGCTCTTCCGATCT- NNNNN-GACTACHVGGGTATCTAATCC |
| 2nd PCR | 1µl of 10x Ex Buffer, 0.8µl of 2.5mM dNTPs, 0.5µl of 10µM forward and reverse primers, 2µl of the PCR product (5ng/µl), 0.1µl of ExTaq HS^*1,^ 5.1µl of nuclease free water | 2 min denaturation step at 94ºC, followed by 10 cycles of 30 s at 94ºC, 60ºC for 30 s, and a final extension at 72ºC for 5 min | F: AATGATACGGCGACCACCGAGATCTACAC-Index2^*2^- ACACTCTTTCCCTACACGACGC  R: CAAGCAGAAGACGGCATACGAGAT-Index1^*2^- GTGACTGGAGTTCAGACGTGTG |

*1: Takara Bio Inc., Shiga, Japan

*2: Sample identification array
