# Supplementary material for: Sulfated vizantin causes detachment of biofilms composed mainly of the genus Streptococcus without affecting bacterial growth and viability
Source: BMC Microbiol. 2020 Nov 25;20:361. doi: 10.1186/s12866-020-02033-w (PMC7687742; doi:10.1186/s12866-020-02033-w)
Supplement: Supplementary file 7 — Additional file 7: Table S2 16S rRNA primers used in this study. [file 12866_2020_2033_MOESM7_ESM.docx]

**Table S2** 16S rRNA primers used in this study.

| Targeted strain | Sequence (5'→3') | Reference |
| --- | --- | --- |
| *S. salivarius* | F: GAGTTGCGAACGGGTGAGTA  R: GGGTCATCCATTGTTATGCGG | This study |
| *S. oralis* | F: CCGCATAAGAGTAGATGTTG  R: TATGTATCGTTGCCTTGGT | 49 |
| *S. mitis* | F: GATTGTAGGCTGCAACTCGC  R: GGCTGGCTCCTTACGGTTAC | This study |
| *S. gordonii* | F: GCTTGCTACACCATAGACT  R: CCGTTACCTCACCTACTAG | 49 |
| *S. mutans* | F: AGTCGTGTTGGTTCAACGGA  R: TAAACCGGGAGCTTGATCGG | 47 |
| *S. sanguinis* | F: AGTTGCCATCATTGAGTTG  R: GTACCAGCCATTGTAACAC | 49 |
| *Streptococcus* | F: TCGGATCGTAAAGCTCTGTTGTA  R: GGACAACGCTCGGGACCTAC | *1 |

*1: Method for measuring the number of oral streptococci and a pcr primer-probe set used for the same. (Publication number: US20080182265A1, JP2008206516A).
