# Supplementary material for: Sulfated vizantin causes detachment of biofilms composed mainly of the genus Streptococcus without affecting bacterial growth and viability
Source: BMC Microbiol. 2020 Nov 25;20:361. doi: 10.1186/s12866-020-02033-w (PMC7687742; doi:10.1186/s12866-020-02033-w)
Supplement: Supplementary file 8 — Additional file 8: Table S3 Primer sequences used for analyzing the genes associated with biofilm formation by real-time PCR. [file 12866_2020_2033_MOESM8_ESM.docx]

**Table S3** Primer sequences used for analyzing the genes associated with biofilm formation by real-time PCR.

| Targeted strain | Genetic location | Sequence (5'→3') | Reference |
| --- | --- | --- | --- |
| *S. salivarius* | *gtfJ* | F: AAGAAGCTGTTGTACCTGAATTTGA  R: CTTGCTACTGTCTTGGGAGGA | This study |
| *S. salivarius* | *gtfK* | F: GCTGCAGCAGTAGAGGTTGA  R: ACTTCTCAGTCGTTGGAGCG | This study |
| *S. oralis* | *gtfR* | F: CCTTTTGATGGCTTGGTGGC  R: ATTTTGCGCTGAACCTGCTG | This study |
| *S. sanguinis* | *gtfP* | F: GCCCAAATTCTCAACCGTTAC  R: ATCTTGCCCTTGACTTGGTAG | 55 |
| *S. gordonii* | *gtfG* | F: AGAGCGTTTGCCAGAACCA  R: CCAACACATCGTCATCATGCT | 50 |
| *S. mutans* | *gtfB* | F: GATCAAGATGTTCGCGTTGC  R: ACACATACTGCGGTGCCATT | 53 |
| *S. mutans* | *gtfC* | F: GATCAAGAAGCGGCTGGTTT  R: ACATGACGCGTGAATCAAGG | 53 |
| *S. mutans* | *gtfD* | F: ATTAAATATGCCGCCGGTGG  R: ATGCGCAGTCCCCATATTGA | 53 |
| *S. oralis* | *comE* | F: AGGCAACAGGAAAAGTCCGT  R: ACGAATGAGCTGAGCAACCT | This study |
| *S. oralis* | *luxS* | F: GCAACCGAACGAAGACTCAA  R: TCATCCCGTCGATTCGAGTA | This study |
| *S. sanguinis* | *comE* | F: GGACAGCGATGATTTTCGTA  R: AGCTCTCATAAACTTCGGATTG | 51 |
| *S. gordonii* | *comD* | F: AAATGCACATCTTAATAGCTTTGCTAGT  R: CATATTGTTCACGAGCAGACTTCAG | 50 |
| *S. gordonii* | *luxS* | F: TGATTGCTCCCCTTTTGGCT  R: GCGATCTCTTCGAGGCATGA | This study |
| *S. mutans* | *comE* | F: GAGTTCTCCCACCGCATTGA  R: ACCATTCTTCTGGCTGTTTTCC | 52 |
| *S. mutans* | *luxS* | F: ACTGTTCCCCTTTTGGCTGTC  R: AACTTGCTTTGATGACTGTGGC | 54 |
