# Supplementary material for: Sulfated vizantin causes detachment of biofilms composed mainly of the genus Streptococcus without affecting bacterial growth and viability
Source: BMC Microbiol. 2020 Nov 25;20:361. doi: 10.1186/s12866-020-02033-w (PMC7687742; doi:10.1186/s12866-020-02033-w)
Supplement: Supplementary file 9 — Additional file 9: Table S4 Primer sequences used for analyzing the genes associated with bacterial adhesion by real-time PCR. [file 12866_2020_2033_MOESM9_ESM.docx]

**Table S4** Primer sequences used for analyzing the genes associated with bacterial adhesion by real-time PCR.

| Targeted strain | Genetic location | Sequence (5'→3') | Reference |
| --- | --- | --- | --- |
| *S. oralis* | PAc protein homolog/SpaA protein homolog | F: AGCCTCACTTAACCGTGAGC  R: CCTGCACCGTACCAAGAGTT | This study |
| *S. mitis* | *aliC* | F: AACACTTGGAACGGAGAATG  R: GCCCTTTGTTATACCTAGATGTTTC | 58 |
| *S. mitis* | *cps2K* | F: CTTCCTAAAGATACCAAGCAACT  R: CCTTGAATAGAGCTTTGACGA | 59 |
| *S. mitis* | *psaA* | F: GACCCACACGAATACGAACC  R: TAAACCAAGCATTGCCACCT | 56 |
| *S. mitis* | *pspC* | F: TCCAATTAGATAGAAGAAAACATACCC  R: TGCTCAAAAGCTGCGTCTAA | 56 |
| *S. mitis* | *RfbC* | F: TTGATCTACGCGAGGGTGAA  R: TGTTTATCTGCTTCTGAAACCTCT | 59 |
| *S. sanguinis* | *ciaR* | F: AAAGGACGGTTTCCAAGTGC  R: TTACCAGCGCGTTTCAAGAG | 55 |
| *S. gordonii* | *sspA* | F: TCCTGACAAACCTGAGACACC  R: TTTAACTTTCAGAGCTTAGTTGCTTTC | 60 |
| *S. gordonii* | *sspB* | F: TCCTGACAAACCTGAGACACC  R: CATCAAAGATGAAACAAGTCTAAGC | 60 |
| *S. gordonii* | *cshA* | F: CAGACGATGCAACCCCTATT  R: TAACGGTCAAGGTCACCACA | 60 |
| *S. gordonii* | *abpA* | F: TGATGCAGTTGAAGGTGGAA  R: TAGCTGCACCAACACGTTTC | 60 |
| *S. gordonii* | *abpB* | F: CAAAAACTCCGGAAAAACCA  R: GGAGCTTGACTCGGTTCTTG | 60 |
| *S. gordonii* | *gspB* | F: TGCTGCAAATGGTGGAGTCT  R: CTTCCCCACCGGTATCGTTT | This study |
| *S. mutans* | *gbpB* | F: AGTGCTTCGACAGCTGCTGT  R: AACCGCCATCATTCCATACA | 53 |
| *S. mutans* | *gbpC* | F: TCCTCCAGTGACACCACCAA  R: AAAGTAGCCGCAGCAAATGC | 53 |
| *S. mutans* | *gbpD* | F: TTCCGCTTCTAGCCAGCAAT  R: AATGCGTCGGCTATCGATGT | 53 |
| *S. mutans* | *ftf* | F: AAATATGAAGGCGGCTACAACGC  R: CTTCACCAGTCTTAGCATCCTGAA | 54 |
| *S. mutans* | *pac* | F: AGCTGGAGAGACAAATGGTTCAT  R: GACACCAGCAGACTTAGCATCTT | 57 |
| *S. mutans* | *spaP* | F: TCCGCTTATACAGGTCAAGTTG  R: GAGAAGCTACTGATAGAAGGGC | 54 |
| *S. mutans* | *srtA* | F: GAAGCTTCCTGTAATTGGCG  R: TTCATCGTTCCAGCACCATA | 54 |
